# Classification of different pineapple varieties grown in Malaysia based on volatile fingerprinting and sensory analysis

**DOI:** 10.1186/s13065-018-0505-3

**Published:** 2018-12-19

**Authors:** Ola Lasekan, Fatma Khalifa Hussein

**Affiliations:** Department of Food Technology, University Putra Malaysia, 43400 UPM Serdang, Malaysia

**Keywords:** Pineapple varieties, Volatile fingerprinting, PCA, HCA, Sensory evaluation, GC-O

## Abstract

**Background:**

Pineapple is highly relished for its attractive sweet flavour and it is widely consumed in both fresh and canned forms. Pineapple flavour is a blend of a number of volatile and non-volatile compounds that are present in small amounts and in complex mixtures. The aroma compounds composition may be used for purposes of quality control as well as for authentication and classification of pineapple varieties.

**Results:**

The key volatile compounds and aroma profile of six pineapple varieties grown in Malaysia were investigated by gas chromatography–olfactometry (GC-O), gas-chromatography–mass spectrometry and qualitative descriptive sensory analysis. A total of 59 compounds were determined by GC-O and aroma extract dilution analysis. Among these compounds, methyl-2-methylbutanoate, methyl hexanoate, methyl-3-(methylthiol)-propanoate, methyl octanoate, 2,5-dimethyl-4-methoxy-3(2H)-furanone, δ-octalactone, 2-methoxy-4-vinyl phenol, and δ-undecalactone contributed greatly to the aroma quality of the pineapple varieties, due to their high flavour dilution factor. The aroma of the pineapples was described by seven sensory terms as sweet, floral, fruity, fresh, green, woody and apple-like.

**Conclusion:**

Inter-relationship between the aroma-active compounds and the pineapples revealed that ‘Moris’ and ‘MD2’ covaried majorly with the fruity esters, and the other varieties correlated with lesser numbers of the fruity esters. Hierarchical cluster analysis (HCA) was used to establish similarities among the pineapples and the results revealed three main groups of pineapples.

## Background

Pineapple (*Ananas comosus* L. Merr) which is one of the most popular exotic fruits in the world trade is widely distributed in tropical regions such as the Philippines, Thailand, Malaysia and Indonesia. In 2016, the global pineapple production was estimated at 24.78 million metric tons with Costa Rica (2930.66 metric tons), Brazil (2694.56 metric tons), Philippines (2612.47 metric tons), India (1964 metric tons),Thailand (1811.59 metric tons, and Nigeria (1591.28 metric tons) as the top five pineapple producers in the world [1]. Other important producers are: Indonesia, China, India, Mexico, and Colombia [2]. Malaysia is part of a new group of pineapple-producing countries. Malaysia exported approximately 20,000 tons of fresh pineapples annually [2]. The main pineapple varieties grown in Malaysia are: ‘Moris’, ‘N36’, ‘Sarawak’, ‘Gandul’, ‘Yankee’, ‘Josapine’, ‘Maspine’, and most recently ‘MD2’. Some of these varieties such as N36 and Josapine were locally developed for the local fresh fruit market.

Pineapple is highly relished for its attractive sweet flavour and it is widely consumed in both fresh and canned forms [3]. Pineapple flavour is a blend of a number of volatile and non-volatile compounds that are present in small amounts and in complex mixtures [4]. The volatile constituents of pineapples have been studied extensively and more than 280 compounds have been reported [4, 5]. Aroma chemicals are organic compounds with defined chemical structures. They are generated by organic or bio-catalytic synthesis or isolated from microbial fermentations [4]. There are many pathways involved in volatile biosynthesis starting from lipids [6], amino acids [7], terpenoids [8] and carotenoids [9]. Once the basic skeletons are produced via these pathways, the diversity of volatiles is achieved via additional modification reactions such as acylation, methylation, oxidation/reduction and cyclic ring closure [6]. As the content of aroma compounds in pineapple depends on many factors such as the climatic and geographical origin [10], varieties [11], different stages of ripening [12], and postharvest storage conditions [13], the aroma compounds composition may be used for purposes of quality control as well as for authentication and classification of pineapple varieties.

Fingerprinting techniques, based on chemical composition and multivariate statistical analysis have been used in characterising or classifying wines according to origin, quality, variety and type [14, 15]. It was also used in the authentication of green-ripe sea-freighted and air-freighted pineapple fruits harvested at full maturity [16]. Application of untargeted fingerprinting techniques as a means of gaining insight into the reaction complexity of a food system has received tremendous interest among researchers [17]. Fingerprinting is defined as a more unbiased and hypothesis-free methodology that considers as many compounds as possible in a particular food fraction [18]. Fingerprinting doesn’t concentrate on a specifically known compound, rather it allows for an initial fast screening to detect differences among samples. Meanwhile, chemometric techniques such as principal component analysis (PCA) and hierarchical cluster analysis (HCA) are employed in the analysis of generated data. PCA is often complemented with HCA to explore data sets obtained by gas chromatography. This method has been used in the classification of wines based on their volatile profiles [19]. Multivariate techniques of data analysis represent a useful statistical tool to differentiate between different fruit varieties [20]. Also, this chemometric approach has been used to classify muskmelon [21], tomato fruit [22], and citrus juice [20].

Although much work has been done on volatile fingerprinting in apple fruits [23], and grape fruits [24], there has been no systematic study on volatile fingerprinting of fresh pineapple fruits grown in Malaysia. The purpose of this study were: (1) to identify and quantify the volatile compounds in six different varieties of pineapples grown in Malaysia (Moris, Maspine, MD2, N36, Josapine and Sarawak) and (2) apply fingerprinting technique to determine which volatile compounds may be potential markers for pineapple varieties grown in Malaysia.

## Results and discussion

### Sensory evaluation

The aroma qualities of the six different pineapple varieties were elucidated by ten trained panellists. The obtained relative standard deviation from the mean aroma quality intensities varied within the range of 1.2–5.9% depending on the pineapple variety and the aroma quality. The details of the aroma qualities of the pineapples are listed in Table 1. Results of the aroma qualities revealed significant differences (p < 0.05) among varieties for all attributes. For instance, while pineapple ‘MD2’ presented the highest intensities for sweetness (8.62), floral (6.88) and apple-like (8.31) attributes, ‘Moris’ produced the highest intensities for fruity (6.83) and fresh (7.31) attributes, respectively. On the other hand, ‘Sarawak’ had the strongest woody (7.46) and green (7.62) attributes. The other pineapple varieties (‘Josapine’, ‘N36’ and ‘Maspine’) produced varied aroma responses. ‘Josapine’ had strong sweet and woody attributes with relatively low floral aroma. ‘Maspine’ exhibited strong sweet and green aroma notes. ‘N36’ had strong sweet and woody aroma, respectively.

**Table 1:** The mean scores and relative standard deviation of the seven aroma-attributes for the six pineapple varieties grown in Malaysia

Fruit			Mean values				
	Sweet (RSD %)	Floral (RSD %)	Fruity (RSD %)	Fresh (RSD %)	Green (RSD %)	Woody (RSD %)	Apple-like (RSD %)
Moris	8.50^b^ (2.8)	5.67^b^ (4.0)	6.83^a^ (2.2)	7.31^a^ (4.5)	3.85^e^ (4.8)	5.63^d^ (5.9)	6.81^b^ (5.7)
Maspine	6.81^e^ (2.6)	2.56^f^ (3.1)	4.40^f^ (1.2)	6.75^b^ (4.9)	6.00^b^ (5.6)	4.00^f^ (4.8)	6.15^c^ (5.3)
MD2	8.62^a^ (2.9)	6.88^a^ (3.7)	6.40^b^ (1.1)	6.05^c^ (4.1)	2.57^f^ (3.4)	5.15^e^ (3.1)	8.31^a^ (2.6)
N36	7.82^d^ (3.3)	4.66^c^ (4.1)	5.13^e^ (4.3)	4.75^e^ (4.6)	5.26^c^ (4.7)	6.05^c^ (3.0)	4.15^e^ (3.4)
Josapine	8.01^e^ (4.0)	3.58^d^ (3.0)	5.05^d^ (3.7)	5.35^d^ (5.5)	4.50^d^ (5.3)	6.91^b^ (5.0)	5.34^d^ (4.5)
Sarawak	6.45^f^ (2.5)	3.05^e^ (2.7)	5.52^c^ (1.6)	4.54^f^ (2.3)	7.62^a^ (4.4)	7.46^a^ (3.7)	3.56^f^ (2.1)

Superscripts with different letters are significantly (p < 0.05) different

To have an insight into the reasons behind this observation, the different pineapple varieties were subjected to AEDA and GC-O.

### Characterization of aroma-active compounds by GC-O analysis

A total of 59 volatile compounds were detected in the six different pineapple varieties grown in Malaysia (Table 2). The details are listed in Table 2. Pineapple ‘Moris’ had the highest number of compounds with a total of 31 compounds and this was followed by ‘MD2’ with 27 aroma-active compounds. The next were ‘N36’, ‘Maspine’, and ‘Sarawak’ which produced 24, 20 and 18 aroma-active compounds respectively. ‘Josapine had the least number (16) of aroma-active compounds. Some of the compounds detected were methyl-2-methylbutanoate, dimethyl malonate, methyl-2-methyl acetoacetate, methyl-2-hydroxy-2-methylbutanoate, methyl hexanoate, ethyl isohexanoate, methyl-2-methylhexanoate, methyl-3-(methylthiol)-propanoate, ethyl hexanoate, y-lactone, 2,5-dimethyl-4-hydroxy-3(2H)-furanone, methyl-3-hydroxyhexanoate, 2,5-dimethyl-4-methoxy-3(2H)-furanone, methyl octanoate, methyl-(4E)-octenoate, 2,4-dihydroxy-2,5-dimethyl-3(2H)-furanone. Among the aforementioned compounds, 12 aroma-active compounds with flavour dilution (FD) ≥ 16 were identified as key odorants through the application of the aroma extract dilution analysis (AEDA) (Table 2). For all the pineapple varieties, the highest FD factor was attributed to methyl-2-methylbutanoate (FD, 1024), methyl hexanoate (FD, 128) and 2,4-dihydroxy-2,5-dimethyl-3(2H)-furanone (DMHF) (FD, 128), respectively.

**Table 2:** Detected aroma compounds with retention index and mean concentration (µg/kg fresh fruit) found in each pineapple varieties grown in Malaysia

No	Compound^a^	Aroma-quality^b^	Moris	Maspine	MD2	N36	Josapine	Sarawak	RI on TG-5 ms
C1	Methyl-2-methylbutanoate	Apple-like	103 ± 8.5	–	–	–	–	–	771 [770] [31]
C2	2-Hexanol	Winey	2.1 ± 0.0	–	–	–	–	1.0 ± 0.0	780 [786] [32]
C3	3-Methylbutanoic acid	Cheesy	–	–	21.0 ± 1.5	–	–	–	792
C4	Methyl butyl acetate	Banana	8.0 ± 1.0	–	–	–	–	–	812
C5	Methyl-2-methylpentanoate	Fruity	7.3 ± 1.2	–	–	–	–	6.7 ± 0.1	823 [nf]
C6	Gamma-butyrolactone	Milky	–	–	3.0 ± 0.1	–	–	–	837
C7	Dimethyl malonate	Fruity	48.2 ± 3.5	–	2.0 ± 0.0	–	–	2.0 ± 0.0	843 [nf]
C8	Ethyl-2,3-dimethylbutanoate	Fruity	1.5 ± 0.0	–	–	–	–	–	856 [856] [32]
C9	Methyl-2-methyl acetoacetate	Fruity	156.1 ± 12.0	–	–	13.0 ± 1.5	–	–	868 [nf]
C10	Methyl-2/3-hydroxy-2/3-methylbutanoate	Fruity	86.0 ± 6.5	–	7.0 ± 0.1	–	–	–	877
C11	Methyl hexanoate	Fruity	397 ± 15.0	tr	44.0 ± 2.1	19.0 ± 0.1	tr	32.0 ± 1.0	884
C12	Ethyl isohexanoate	Pineapple	13.0 ± 1.0	–	–	–	–	–	920
C13	Methyl-2-methylhexanoate	Fruity	–	–	8.0 ± 0.1	–	–	–	931
C14	Methyl-3-(methylthiol)-propanoate	Sulphurous	307 ± 9.7	–	28.7 ± 1.0	–	–	17.0 ± 0.1	936
C15	Hexanoic acid	Fatty	–	–	12.4 ± 0.1	–	–	–	974 [975] [32]
C16	(E)-β-Ocimene	Sweet/herbal	4.0 ± 0.0	–	1.0 ± 0.0	–	2.0 ± 0.0	1.0 ± 0.0	976
C17	Methyl-3-hydroxy-4-methylpentanoate	Fruity	65.0 ± 5.6	–	–	–	–	–	983
C18	Ethyl hexanoate	Fruity	13.0 ± 1.2	–	–	–	–	1.0 ± 0.0	984 [1002] [32]
C19	Gamma-lactone	Creamy	202.0 ± 9.7	–	–	–	11.0 ± 0.1	5.0 ± 0.1	986 [986] [32]
C20	Delta-lactone	ND	221 ± 11.0	–	–	15.1 ± 1.2	9.0 ± 0.1	5.6 ± 0.1	1006
C21	2,5-Dimethyl-4-hydroxy-3(2H)-furanone	Strawberry	55.0 ± 3.4	9.0 ± 1.0	1.5 ± 0.0	1.2 ± 0.0	54.2 ± 2.0	6.0 ± 0.1	1022
C22	Methyl-3-hydroxyhexanoate	Fruity	–	–	11.2 ± 0.1	–	–	–	1047
C23	2,5-Dimethyl-4-methoxy-3(2H)-furanone	Roasty/sweet	–	–	7.4 ± 0.1	–	–	–	1055
C24	Methyl octanoate	Fruity	101.0 ± 8.0	–	3.0 ± 0.0	–	–	4.0 ± 0.1	1083
C25	Methyl (4E)-4-octenoate	Fruity	30.0 ± 3.0	–	–	–	–	–	1091
C26	3-Octyl acetate*	Herbal/green	–	–	2.0 ± 0.0	–	–	–	1118 [1119] [32]
C27	2,4-Dihydroxy-2,5-dimethyl-3(2H)-furanone	Fruity	2.0 ± 0.0	3.2 ± 0.1	4.3 ± 0.1	–	–	–	1173
C28	Octanoic acid	Rancid	5.0 ± 0.1	2.0 ± 0.0	2.1 ± 0.0	–	–	–	1174
C29	Gamma-octalactone	Coconut-like	86.2 ± 4.0	–	–	–	–	–	1184
C30	Delta-octalactone	Creamy	11.0 ± 1.5	–	3.5 ± 0.0	3.0 ± 0.1	11.0 ± 0.1	7.0 ± 0.1	1205
C31	Copaene	Woody	40.1 ± 3.9	–	12.0 ± 1.2	–	3.0 ± 0.1	–	1221
C32	Methyl decanoate	Floral	4.0 ± 0.1	–	–	–	–	–	1282
C33	2-Methoxy-4-vinyl phenol	Smoky	–	4.0 ± 0.1	2.0 ± 0.0	18.0 ± 1.0	–	–	1293
C34	Decanoic acid	Sweaty	–	2.0 ± 0.0	2.0 ± 0.0	–	2.0 ± 0.0	–	1372
C35	Methyl-5-acetoxy octanoate	Wine-like	5.0 ± 0.1	–	–	–	–	–	1385
C36	gamma-Farnesene	ND	–	–	2.7 ± 0.0	–	–	–	1453
C37	Delta-undecalactone*	Coconut-like	–	–	2.0 ± 0.1	–	4.1 ± 0.1	–	1483 [1488] [33]
C38	Germacrene	Woody	–	–	1.0 ± 0.0	–	–	–	1515 [1502] [33]
C39	Globulol	Floral	–	–	2.0 ± 0.1	–	–	–	1530
C40	(-)-Spathulenol	Earthy	19.0 ± 1.0	–	8.0 ± 1.5	–	–	–	1536
C41	Dodecanoic acid	Sweaty/soapy	7.0 ± 0.1	–	–	3.0 ± 0.1	–	–	1570
C42	Gamma-dodecalactone	Fruity	–	–	–	1.0 ± 0.0	–	–	1582 [1587] [32]
C43	(Z)-7-Tetradecenal	ND	–	1139 ± 34.0	–	–	–	–	1609
C44	Pentadecanal*	Fresh/waxy	18.1 ± 1.0	–	–	4.0 ± 0.1	–	–	1701 [1712] [36]
C45	3,5-Dimethoxy-4-hydroxycinnamaldehyde	Cocoa-like	–	3.0 ± 0.1	–	–	–	–	1788
C46	Pentadecanoic acid	Waxy	–	3.0 ± 0.1	–	2.0 ± 0.0	–	1.0 ± 0.0	1869
C47	Methylhexadecanoate*	Waxy	–	2.6 ± 0.0	–	–	–	1.0 ± 0.0	1878[1878] [32]
C48	Methyl-(2E)-2-hexadecenoate	ND	–	8.0 ± 0.1	–	–	–	–	1886
C49	Ethyl hexadecanoate	Waxy	–	4.0 ± 0.1	–	–	1.0 ± 0.0	–	1928
C50	Hexadecanoic acid*	Waxy	–	51.7 ± 3.2	255.0 ± 9.0	5.0 ± 0.1	393.0 ± 11.2	2.0 ± 0.0	1968 [1970] [32]
C51	9-Hexadecenoic acid	Waxy	–	2.0 ± 0.0	–	–	–	–	1976
C52	Octadecanal	Fatty/greasy	–	–	–	21.0 ± 1.5	–	–	1999 [2002] [32]
C53	Eicosane	ND	105.1 ± 9.0	2.0 ± 0.0	–	14.0 ± 2.0	2.0 ± 0.0	16.0 ± 1.0	2009
C54	Heptadecanoic acid*	Waxy	–	4.0 ± 0.1	–	–	3.0 ± 0.1	–	2067 [2067] [32]
C55	Octadecanoic acid*	Pungent	–	149.0 ± 9.0	1.0 ± 0.0	69.0 ± 5.1	–	89.0 ± 7.0	2167 [2167] [32]
C56	Ethyl octadecanoate*	Waxy	–	46.0 ± 3.0	–	–	2.0 ± 0.0	–	2177 [2174] [33]
C57	(Z,Z)-9,12-Octadecadienoic acid*	Waxy	–	37.0 ± 2.1	–	–	37.0 ± 4.0	16.0 ± 1.5	2183 [2183] [36]
C58	Ethyl oleate*	Fatty	–	89.0 ± 6.5	–	–	57.0 ± 2.0	–	2185 [2180] [32]
C59	Geranyl geraniol	Floral	11.0 ± 0.1	–	–	–	–	–	2192

– Odorant not detected

*ND* not detectable

*tr* Trace (< 1.0 µg/kg), [RI_lit_]^35^; Scheidig et al. [31], [RI_lit_]^36^; NIST [32], [RI_lit_]^37;^ El-Sayad [33]

^a^Compounds were identified by comparing their retention indices on the TG-5 ms column, their mass spectra, and odour nuances with the respective data of the reference odorants

^b^Aroma-quality perceived by panellists during olfactometry

* Compounds tentatively identified with the MS database and retention index

Meanwhile, methyl-2-methylbutanoate which exhibited the highest FD factor had a bigger influence on the aroma profile of pineapple ‘Moris’. It was however, not detected in the other varieties. On the other hand, methyl hexanoate and DMHF contributed significantly to the aroma profiles of the different pineapple varieties. This observation was similar to those of Zheng et al. [3]. For instance, the FD factors of methyl hexanoate in the different pineapple varieties were 64, 128, 64, 32 and 16 corresponding to ‘Moris’, ‘MD2’, ‘N36’, ‘Josapine’ and ‘Sarawak’. 2,4-Dihydroxy-2,5-dimethyl-3(2H)-furanone had greater influence on the aroma profiles of “Moris’ ‘Maspine’ and ‘MD2’ with a corresponding FD factors of 16, 64 and 128, respectively. In addition, aroma-active compounds with relatively high FD factors such as δ-octalactone, 2-methoxy-4-vinyl phenol, methyl octanoate and hexadecanoic acid had appreciable influence on the aroma profile of the pineapple varieties (Table 2).

### Quantitation of aroma-active compounds

The detected aroma-active compounds and their mean concentrations were listed in Table 3. Most of the aroma-active compounds were branched esters. Recently, Steingrass et al. [12, 21] also reported that esters were the main volatile compounds in fresh pineapple, which is in agreement with our findings. In addition, several other groups of compounds such as ketones, alcohols, terpenes, lactones and acids were detected in the different pineapple varieties. Branched esters such as methyl-2-methyl butanoate, methyl-2-methyl pentanoate, ethyl-2,3-dimethylbutanoate, methyl-2-methyl acetoacetate, methyl-2-hydroxy-2-methylbutanoate, methyl-3-(methylthiol)-propanoate, methyl-3-hydroxy-4-methylpentanoate, methyl hexanoate, and methyl-3-hydroxyhexanoate were the most abundant compounds. Among these compounds, methyl-3-(methylthiol)-propanoate (307 ± 9.7 µg/kg) methyl-2-methylbutanoate (103 ± 8.5 µg/kg), methyl-2-hydroxy-methylbutanoate (86.0 ± 6.5 µg/kg), methyl-3-hydroxy-4-methyl pentanoate (65.0 ± 5.6 µg/kg), methyl hexanoate (397 ± 15 µg/kg) and methyl-2-methyl acetoacetate (156.1 ± 12.0 µg/kg) produced higher concentrations than other esters in the pineapple varieties (Table 3). However, research to determine the mechanism by which these esters are generated has been limited. The primary enzyme believed to be responsible for ester production is the alcohol acyltransferase (AAT), which was first isolated from ‘Chandler’ fruit [25].

**Table 3:** Detected aroma compounds with their flavour dilution (FD) factors in each pineapple varieties (Moris, Maspine, MD2, N36, Josapine and Sarawak) grown in Malaysia

No	Compound^a^	Aroma-quality^b^	Moris	Maspine	MD2	N36	Josapine	Sarawak	RI on TG-5 ms
1	Methyl-2-methylbutanoate	Fruity	1024	–	–	–	–	–	771
2	2-Hexanol	Winey	2	–	–	–	–	2	780
3	3-Methylbutanoic acid	Cheesy	–	–	2	–	–	–	792
4	Methyl butyl acetate	Banana	2	–	–	–	–	–	812
5	Methyl-2-methylpentanoate	Fruity	4	–	–	–	–	2	823
6	Gamma-butyrolactone	Weak, milky	–	–	2	–	–	–	837
7	Dimethyl malonate	Fruity	8	–	2	–	–	2	843
8	Ethyl-2,3-dimethylbutanoate	Fruity	8	–	–	–	–	–	856
9	Methyl-2-methyl acetoacetate	Fruity	8	–	–	8	–	–	868
10	Methyl-2/3-hydroxy-2/3-methylbutanoate	Fruity	8	–	4	–	–	–	877
11	Methyl hexanoate	Fruity	64	–	128	64	32	16	884
12	Ethyl isohexanoate	Pineapple	8	–	–	–	–	–	920
13	Methyl-2-methylhexanoate	Sulfurous	8	–	4	–	–	2	931
15	Hexanoic acid	Fatty	–	–	2	–	–	–	974
16	(E)-β-Ocimene	Sweet, herbal	2	–	2	–	2	2	976
17	Methyl-3-hydroxy-4-methylpentanoate	Fruity	8	–	–	–	–	–	983
18	Ethyl hexanoate	Fruity	16	–	–	–	–	16	984
19	Gamma-lactone	Creamy	16	–	–	–	16	8	986
20	Delta-lactone	ND	–	–	–	–	–	–	1006
21	2,5-Dimethyl-4-hydroxy-3(2H)-furanone	Strawberry	16	16	–	–	32	16	1022
22	Methyl-3-hydroxyhexanoate	Fruity	–	–	8	–	–	–	1047
23	2,5-Dimethyl-4-methoxy-3(2H)-furanone	Caramel, sweet	–	–	32	–	–	–	1055
24	Methyl octanoate	Fruity	32	–	16	–	–	16	1083
25	Methyl (4E)-4-octenoate	Fruity	8	–	–	–	–	–	1091
26	3-Octyl acetate	Herbal/green	–	–	2	–	–	–	1118
27	2,4-Dihydroxy-2,5-dimethyl-3(2H)-furanone	Fruity	16	64	128	–	–	–	1173
28	Octanoic acid	Rancid	2	2	2	–	–	–	1174
29	Gamma-octalactone	Coconut	4	–	–	–	–	–	1184
30	Delta-octalactone	Creamy	16	–	32	16	16	16	1205
31	Copaene	Woody	8	–	8	–	2	–	1221
32	Methyl decanoate	Floral	2	–	–	–	–	–	1282
33	2-Methoxy-4-vinyl phenol	Smoky	–	16	4	32	–	–	1293
34	Decanoic acid	Sweaty	–	2	2	–	2		1372
35	Methyl-5-acetoxy octanoate	Winey	8	–	–	–	–	–	1385
36	gamma-Farnesene	ND	–	–	–	–	–	–	1453
37	Delta-undecalactone	Coconut	–	–	32	–	16	–	1483
38	Germacrene	Woody	–	–	2	–	–	–	1515
39	Globulol	Floral	–	–	4	–	–	–	1530
40	(-)-Spathulenol	Earthy	8	–	8	–	–	–	1536
41	Dodecanoic acid	Soapy/sweaty	2	–	–	4	–	–	1570
42	y-Dodecalactone	Fruity	–	–	–	2	–	–	1582
43	(Z)-7-Tetradecenal	ND	–	–	–	–	–	=	1609
44	Pentadecanal	Waxy/fresh	4	–	–	4	–	–	1701
45	3,5-Dimethoxy-4-hydroxycinnamaldehyde	Cocoa-like	–	2	–	–	–	–	1788
46	Pentadecanoic acid	Waxy	–	2	–	2	–	2	1869
47	Methylhexadecanoate	Waxy	–	4	–	4	–	4	1878
48	Methyl-(2E)-2-hexadecenoate	ND	–	–	–	–	–	–	1886
49	Ethyl hexadecanoate	Waxy	–	2	–	–	2	–	1928
50	Hexadecanoic acid	Waxy/sweaty	–	4	64	4	32	2	1968
51	9-Hexadecenoic acid	Waxy	–	2	–	–	–	–	1976
52	Octadecanal	Greasy	–	–	–	4	–	–	1999
53	Eicosane	ND	–	–	–	–	–	–	2009
54	Heptadecanoic acid	Waxy	–	2	–	–	2	–	2067
55	Octadecanoic acid	Pungent/sweaty	–	8	2	4	–	8	2167
56	Ethyl octadecanoate	Waxy	–	8	–	–	2	–	2177
57	(Z,Z)-9,12-Octadecadienoic acid	Waxy/sweaty	–	8	–	–	8	4	2183
58	Ethyl oleate	Fatty	–	8	–	–	8	–	2185
59	Geranyl geraniol	Floral	8	–	–	–	–	–	2192

*ND* not detectable, *FD* Flavour dilution factor determined in extract containing the juice volatiles

– odorant not detected

^a^Compounds were identified by comparing their retention indices on the TG-5 ms column, their mass spectra, and odour nuances with the respective data of the reference odorants

^b^Aroma-quality perceived by panellists during olfactometry

Whilst methyl-branched esters such as methyl-2-methyl butanoate, methyl-2-methylpentanoate, etc. are assumed to be derived from branched-chain amino acid catabolism [25], Methyl-3-(methylthiol)-propanoate which exhibited high concentrations in ‘Moris’, ‘MD2’ and ‘Sarawak’ has been attributed to the Stickland reactions of methionine [26]. It is worthy of note that the ethyl derivatives of odd numbered carboxylic acids or branched carboxylic acids such as ethyl-2,3-dimethylbutanoate, ethyl isohexanoate and ethyl hexanoate were more specific and appeared in appreciable amount in pineapple ‘Moris’ only (Table 3). Furthermore, ‘Moris’ was also characterized by several acetates and acetoxy esters such as methyl-2-methyl acetoacetate, methyl butyl acetate, methyl-5-acetoxy octanoate and 3-octyl acetate. The acetates probably resulted from the condensation of acetyl-CoA with alcohols and hydroxyl-fatty acids [25]. Earlier on Steingass et al. [25] postulated that accumulation of acetyl-CoA under anaerobic condition can facilitate the production of both acetates and acetoxylated esters. To corroborate this position, alcohol acetyl transferase (AATs) enzymes’ involvement in the genesis of acetates have been reported in different fruits such as; apples, bananas, pineapples and melon [16]. In addition, there was a marked dominance of the furanones (i.e. 2,5-dimethyl-4-hydroxy-3(2H)furanone; 2,4-dihydroxy-2,5-dimethyl-3(2H)-furanone) and lactones (i.e. y-lactone, δ-lactone, y-octalactone, and δ-octalactone) in ‘Moris’ as compared to the other pineapple varieties. Surprisingly, δ-undecalactone was mainly detected in ‘MD2’ and ‘Josapine’. Lactones which exhibited creamy and coconut-like aroma notes in the pineapple varieties have been identified as most potent odorants in pineapples [27]. The formation of lactones in fruits has been documented. There are two proposed pathways for the formation of lactones [28]. The first pathway is from unsaturated fatty acids to lactones via hydroperoxy fatty acids and monohydroxy fatty acids under the actions of lipoxygenase (LOX) and peroxygenase (PGX). The second pathway is from unsaturated fatty acids to lactones via epoxy fatty acids and dihydroxy fatty acids under the actions of PGX and epoxide hydrolase. 4-Hydroxy-2,5-dimethyl-3(2H)-furanone and its methyl ether 2,5-dimethyl-4-methoxy-3(2H)-furanone are important odorants of many fruits [29]. Whereas, 4-hydroxy-2,5-dimethyl-3(2H)-furanone and its derivatives are synthesized by a series of enzymatic steps in fruits, they are also products of Maillard reaction [30].

### Relationship between pineapple varieties and odour-active compounds

**Fig. 1:**
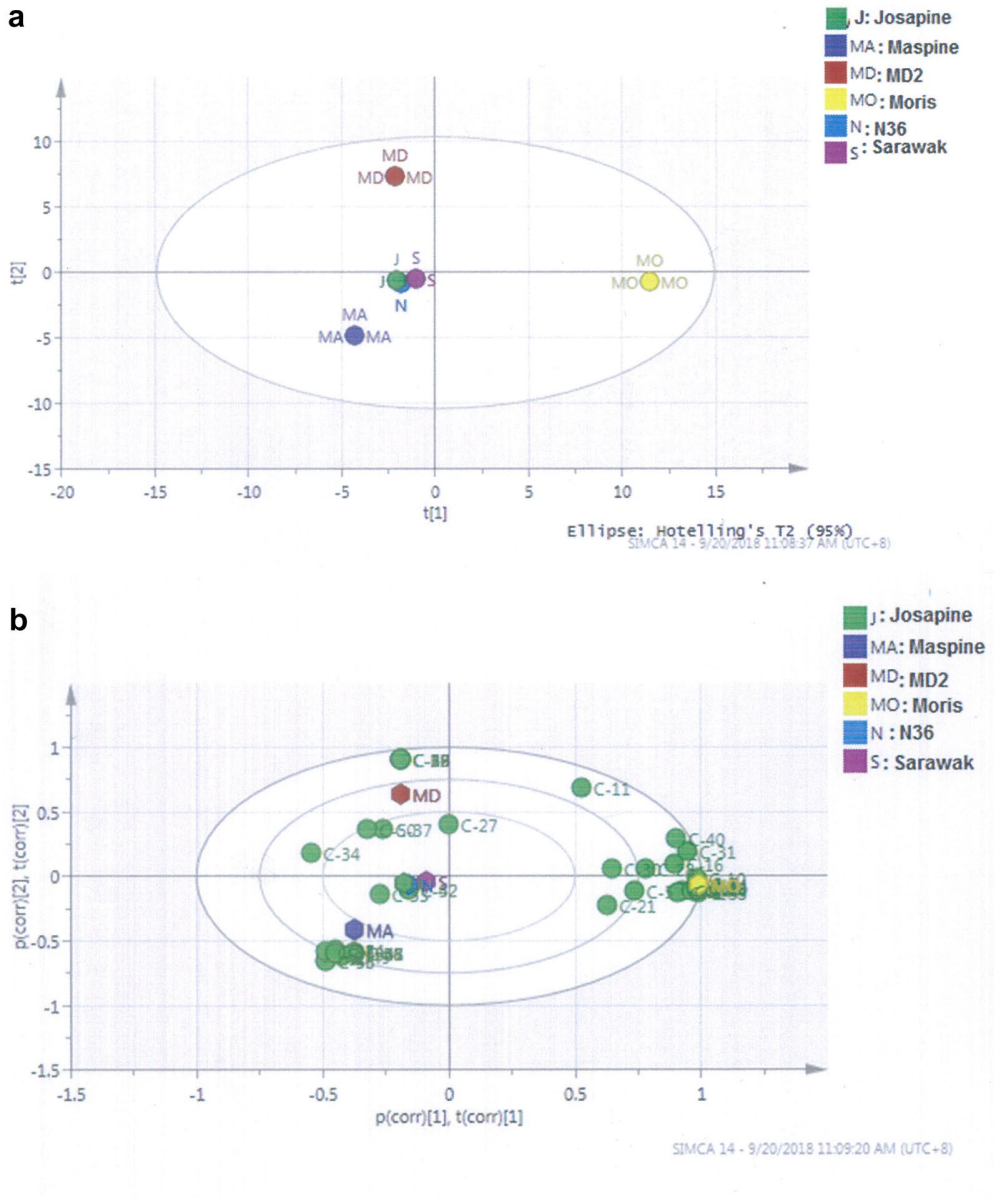
Score scatter PLS-DA and PLS weight plots (**a**, **b**) of the pineapple varieties grown in Malaysia, The PLS-DA plot shows similarities and differences in pineapple varieties while PLS-weight plot reveals the inter-relatedness between the fruits and 97 aroma-active compounds (P1–P97) shown in Table 2

In order to differentiate between the six different pineapples in terms of the aroma-active compounds associated with each variety, principal component analysis (PCA) was used. PCA provides a visual relationship between the pineapple varieties and their aroma-active compounds. This method makes the interpretation of the multivariate analysis easy. A first PCA was performed on the concentration of the 59 volatile compounds (Table 2) analysed in the pineapple varieties. Based on the samples grouping from PCA, a partial least square discriminant analysis (PLS-DA) was established (Fig. 1a). The scatter plot of scores of the first two components (in PLS-DA which explained 95% of the total variance in the data) showed the differences among the six pineapple varieties. The corresponding PLS weight plot (Fig. 1b) revealed the inter-relationship between the aroma compounds and the pineapple varieties.

**Fig. 2:**
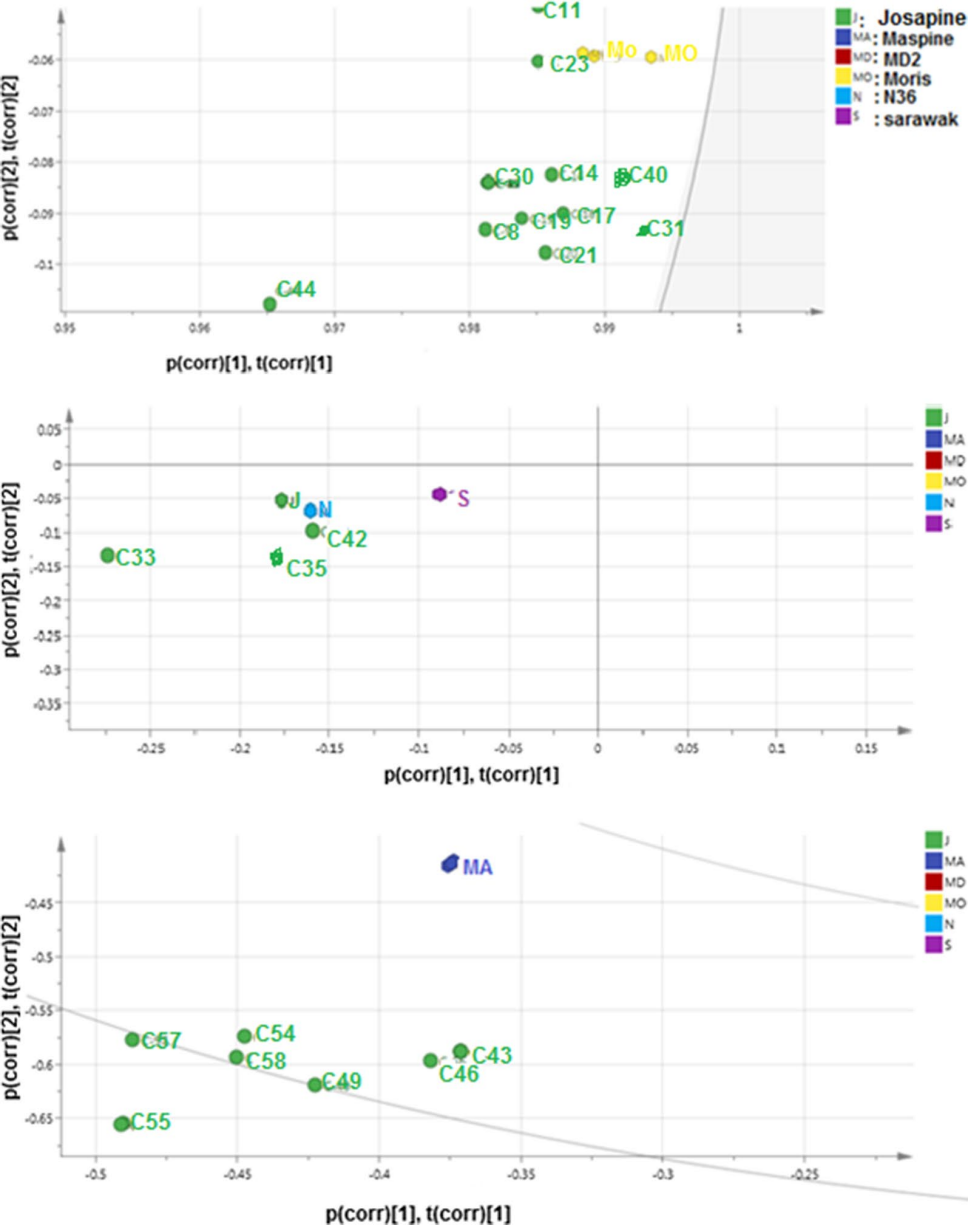
Visualization of PLS weight plot of Fig. 1b. 1, 2 and 3 are aroma compounds correlating with Moris, (yellow), Sarawak, Josapine, N36 and Maspine respectively

Malaysian pineapples were separated according to their varieties (Fig. 1a). Low negative component 1 and high positive component 2 corresponded to pineapple ‘MD2’. The pineapple variety ‘Maspine’ was situated within low negative components 1 and 2, respectively. While pineapple ‘Moris’ was within the area of high positive component 1 and low negative component 2, other varieties such as ‘Sarawak’, Josapine and N36, were all situated at the region of low negative component 1 and low positive component 2.

In addition, the inter-relationship between the aroma-active compounds and the pineapple varieties were carried out by the partial least square (PLS)-weight plot (Fig. 1b). The results revealed that ‘Moris’ covaried with 31 aroma-active compounds, majority of which were the fruity esters with FD ≥ 8 such as methyl-2-methylbutanoate (C1), methyl butyl acetate (C4), ethyl-2,3-dimethylbutanoate (C8), ethyl iso hexanoate (C12), methyl-3-hydroxy-4-methylpentanoate (C17), 2,5-dimethyl-4-hydroxy-3(2H) furanone (C21), methyl octanoate (C25), methyl-5-acetoxy octanoate (C35) and geranyl geraniol (C59) (Table 3) and (Fig. 2). Similarly, ‘Moris’ also covaried with other compounds such as y-octalactone (C29), δ-octalactone (C30), and (-)-spathulenol (C40). On the other hand, ‘Maspine’ was correlated with 2-methoxy-4-vinyl-phenol (C33), (Z)-7-tetradecenal (C43), 3,5-dimethoxy-4-hydroxycinnamaldehyde (C45), pentadecanoic acid (C46), methyl hexadecanoate (C47) and octadecanoic acid (C55) (Fig. 2). In the case of ‘Sarawak’, ‘Josapine’ and ‘N36’, they covaried with ethyl hexanoate (C18)), y-lactone (C42)), methyl octanoate (C24), δ-octalactone (C20), and 2-methoxy-4-viny phenol (C33). However, ‘MD2’ covered with methyl-3(methylthiol)-propanoate (C14), methyl-3-hydroxyhexanoate (C22), 2,4-dihydroxy-2,5-dimethyl-3 (2H)-furanone (C27), δ-undecalactone (C37), (Z)-7-tetradecenal (C43), 3,5-dimetoxy-4-hydroxycinnamaldehyde (C45), methyl hexadecanoate (C47) and decanoic acid (C34).

**Fig. 3:**
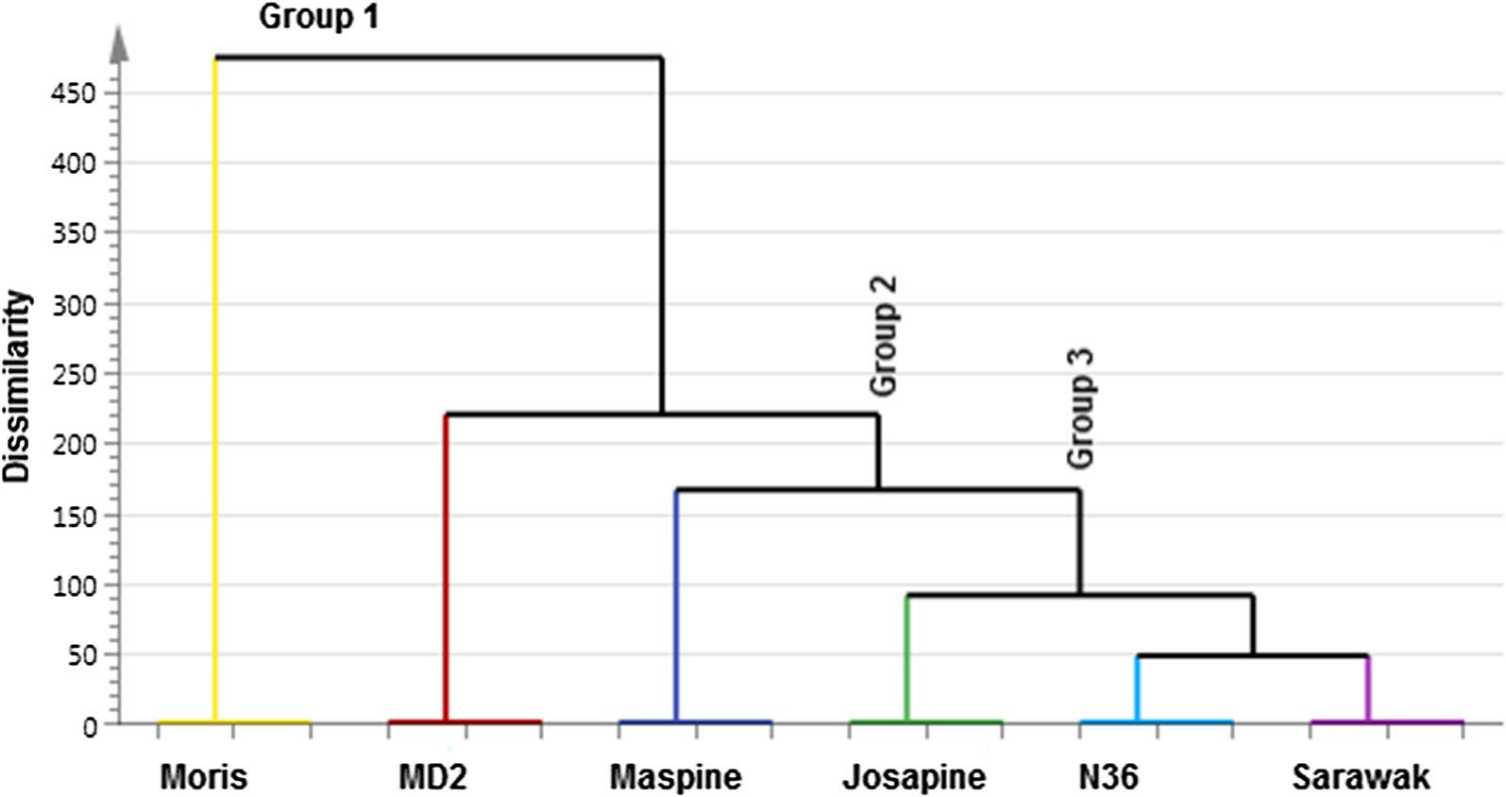
Dendrogram of hierarchical cluster analysis of six pineapple varieties grown in Malaysia

In order to validate the results obtained by PCA analysis, a hierarchical cluster analysis (HCA) was carried out using Ward’s method of agglomeration and Euclidean distances to evaluate similarity between varieties. The test was performed on the complete dataset, thus obtaining the dendrogram in Fig. 3. Three main groups of pineapple varieties were identified by HCA. The first group comprised pineapple ‘Moris’ and ‘MD2’ Fig. 3. This group was characterized by high numbers of aroma-compounds most especially the fruity esters. They contained some of the highly intense aroma-active compounds (FD ≥ 64) such as methyl-2-methyl butanoate, methyl hexanoate, methyl-3-(methylthiol)-propanoate and 2,4-dihydroxy-2,5-dimethyl-3 (2H)-furanone. The second group contained pineapple ‘Maspine’. This group contained the least quantity of fruity esters. The third group included ‘Sarawak’, ‘Josapine’ and ‘N36’. This group contained more of the fatty acid methyl esters.

## Conclusion

Sensory evaluation, GC-O and GC–MS analysis were employed to elucidate the characteristic aroma of six pineapples varieties grown in Malaysia. Application of qualitative descriptive sensory analysis on the six pineapple varieties revealed seven quality terms such as sweet, floral, fruity, fresh, green, woody and apple-like. In addition, 97 aroma-active compounds were identified by GC-O and AEDA in the pineapple varieties. Of this, pineapple ‘Moris’ had the highest numbers of aroma-active compounds with a total of 31 compounds and this was followed by ‘MD2’ with 27 compounds. The next were the ‘N36’, ‘Maspine’, and ‘Sarawak’ which produced 24, 20 and 18 aroma-active compounds, respectively. ‘Josapine’ had the least number of aroma-active compounds (16). In order to address the inter-relationship between the sensory attributes and the aroma compounds, the PLSR analysis was employed. Results of the analysis showed that ‘Moris’ and ‘MD2’ covaried majorly with the fruity esters with higher FD factors. ‘Sarawak’, ‘Josapine’ and ‘N36’ were correlated with fewer fruity esters; they covaried majorly with the lactones. However, the variety ‘Maspine’ was correlated with 2-methoxy-4-vinyl-phenol (C33), (Z)-7-tetradecenal (C43), 3,5-dimethoxy-4-hydroxycinnamaldehyde (C45), pentadecanoic acid (C46), methyl hexadecanoate (C47) and octadecanoic acid (C55), respectively. In addition, hierarchical cluster analysis was used to establish similarities among the pineapples and the results revealed three main groups of pineapples.

## Experimental

### Pineapple fruits

Fresh, fully-ripe pineapples of six different varieties (‘Moris’, ‘Maspine’, ‘MD2’, ‘N36’, ‘Josapine’, and ‘Sarawak’) grown in Johor, Malaysia were obtained from an established farmer. Three fruits of each variety were stored at 8 ± 1 °C and 80–90% relative humidity until analysed. Fruits were selected with similar characteristics of ripening (i.e. pale-yellow skin colour; flat eyes; and degree of Brix), hand-peeled, cored, sliced and cut into small pieces before blending with a Panasonic Food Processor (model PSN-MKF300, Panasonic, Malaysia). One fruit weighed 927–1201 g apart from the crown. The pH and Brix values were 3.49, 3.50, 3.52, 3.54, 3.60, 10.33 ^o^ Brix, 11.45 ^o^ Brix, 12.48 ^o^ Brix, 13.25 ^o^ Brix, 14.01 ^o^ Brix, and 16.50 ^o^ Brix for Sarawak, Maspine, N36, Josapine, Moris and MD2, respectively. At least three separate measurements were carried out for each analysis.

### Chemicals

Pure reference standards of methyl-2-methylbutanoate (98.0%), 2-hexanol (97.0%), 3-methylbutanoic acid (97.5%), methyl butyl acetate (98.0%), methyl-2-methylpentanoate (99.5%), gamma-butyrolactone (98.0%), dimethyl malonate (97.0%), ethyl-2,3-dimethylbutanoate (99.5%), methyl-2-methyl acetoacetate (99.5%), methyl-2-hydroxy-2-methylbutanoate (98.0%), methyl hexanoate (99.5%), methyl-3-(methylthiol)-propanoate (99.5%), hexanoic acid (97.0%), *trans*-β-ocimene (98.0%), methyl-2-methylhexanoate (99.5%), ethyl hexanoate (98.0%), δ-lactone (98.0%), 2,5-dimethyl-4-hydroxy-3(2H)-furanone (99.5%), methyl-3-hydroxyhexanoate (99.5%), 2,5-dimethyl-4-methoxy-3(2H)-furanone (98.0%), methyl octanoate (99.5%), octanoic acid (97.0%), y-octalactone (98.5%), δ-octalactone (98.0%), copaene (97.0%), methyl decanoate (99.5%), 2-methyl-4-vinyl phenol (99.5%), decanoic acid (97.0%), y-farnesene (98.0%), germacrene (98.0%), globulol (98.0%), spathulenol (98.0 5), (*Z*)-7-tetradecenal (97.0%), and octadecanal (99.5%) were purchased from Aldrich, Steinheim, Germany. Gamma-lactone (98.0%) and methyl dodecane (99.5%) were obtained from Parchem, New Rochelle, NY and Achemica Corp. Aigle, Switzerland, respectively. The *n*-alkane standard (C_7_–C_30_) was obtained from Sigma-Aldrich Chemicals Co. (St. Louis, MO). Other chemicals were of analytical grade.

### Isolation of pineapple volatile compounds

The isolation of the pineapple volatile compounds was performed by extracting 300 mL of juice with dichloromethane (300 mL), followed by distillation in vacuum [34]. A similar workup procedure reported earlier [35] was carried out on juice to produce 400 µL extract.

### GC–MS and GC-FID analyses

The extracts were injected into a QP-5050A (Shimadzu, Kyoto, Japan) gas chromatograph equipped with a GC-17A Ver.3, and a flame ionization detector (FID). Two microliters of the extract was vaporized in the injector port maintained at 220 °C in split less mode (1 min). The oven temperature was varied from 50 °C to 250 °C at 15 °C/min, and holding times of 3 and 10 min respectively [36]. A 30–300 m/z mass range was recorded in full-scan mode. The quadrupole ion source and transfer line temperatures were maintained at 150 and 250 °C. respectively and the ionisation energy was set at 70 eV. The column (30 m × 0.25 mm i.d., and 0.25 µm film thickness; 5% diphenyl/95% dimethylpolysiloxane phase; Thermo Scientific, Milan Italy) was a TG-5 ms [36]. The carrier gas was helium at 1.5 mL/min (column-head pressure of 13 psi).

### GC-O analysis

A Trace Ultra 1300 gas chromatograph (Thermos Scientific, Waltham, MA, USA) fitted with a TG-5 ms column (30 m × 0.25 mm i.d., film thickness, 0.25 µm, Thermo Scientific, Milan Italy) and an ODP 3 olfactory Detector Port (Gerstel, Mulheim, Germany), with additional supply of humidified purge air, was operated as earlier reported by Lasekan [35]. The split ratio between the sniffing port and the FID detector was 1:1. Two replicate samples were sniffed by three trained panellists who presented normalized responses, with strong agreement with one another.

### Identification and quantification

Kovats method which employs a mixture of normal paraffin C_7_-C_30_ as external references was used to calculate the linear retention indices [36]. The identification of compounds was as described by Lasekan and Ng [34]. When it was not possible to find appropriate reference standard, a tentative identification was obtained by matching retention index with mass spectral libraries data (WILEY 275, NBS75K). Semi-quantitative data were obtained by relating the peak area of each compound to that of the corresponding standard and were expressed as µg/kg. For compounds tentatively identified, their semi-quantitative data were obtained by relating their peak area to that of octadecane and were expressed as µg/kg octadecane.

### Aroma extracts dilution analysis (AEDA)

The flavor dilution (FD) factors of the aroma-active compounds were evaluated by GC-O using the AEDA approach earlier reported by Lasekan [35]. Each of the obtained dilution was injected into the GC-O. The highest dilution in which an aroma compound was observed is referred to as the flavor dilution (FD) factor of that compound [37].

### Sensory analysis

Sensory analysis was carried out by ten trained panelists (6 females and 4 males) in a sensory laboratory according to the International Standard ISO 8589: [29]. All panelists who have passed screening test as described earlier [34] were recruited from the University Putra Malaysia. Prior to the test, the panelist were taken through 1 h training session with selected aroma compounds such as: ethyl hexanoate (fruity), 2,5-dimethyl-4-hydroxy-3(2H)-furanone (Strawberry), β-damascenone (floral), ethyl isohexanote (pineapple-like), etc. Descriptors used by panelists were determined after three preliminary sensory experiments. Finally, the panelists were asked to evaluate ortho-nasally fresh pineapple juice placed inside glass containers (7 cm × 3.5 cm). Seven aroma attributes (sweet, floral, fruity, fresh, green, woody and apple-like) were obtained. Panelists were asked to score each attribute on a 10-point interval scale with 9 = strong intensity, and 0 = weak with no perception. To aid the sensory analysis, the following reference compounds: ethyl hexanote (fruity), β-damascenone (floral), methyl-3(methylthiol)-propanoate (apple-like), hexanal (green), germacrene (woody), *p*-anisaldehyde (sweet) and (*E,Z*)-3,5-undecatriene (fresh, pineapple-like) were dissolved in water at a concentration of 100 × above their respective threshold values. The fresh pineapple varieties were evaluated in triplicate and the results obtained were averaged.

### Statistical analysis

Analysis of variance (ANOVA) and Duncan’s multiple comparison tests were carried out to establish if statistical differences existed among individual pineapple variety for each sensory attribute at (p < 0.05). Partial least square discriminate analysis (PLS-DA) and PLS-regression coefficient were employed as an exploratory tool to describe and summarise the data by grouping variables that are correlated. The mean concentrations of the 59 aroma-active compounds and the six different pineapple varieties (Table 3) were the data set. The multivariate statistical analyses were performed using the SIMCA-P software (V. 10.0, Umetricus, Umea, Sweden). Principal Components Analysis (PCA) and Hierarchical Cluster Analysis (HCA) using the Software package SPSS Statistics 17.0 (SPSS Inc., Chicago, IL) were also employed.

## Abbreviations

ANOVA: analysis of variance
PCA: principal component analysis
HCA: hierarchical cluster analysis
FD: flavour dilution
